# Enhancing TFEB-Mediated Cellular Degradation Pathways by the mTORC1 Inhibitor Quercetin

**DOI:** 10.1155/2018/5073420

**Published:** 2018-10-28

**Authors:** Yi Huang, Yan Chen, Amanda Marie Shaw, Howard Goldfine, Junqiang Tian, Jiyang Cai

**Affiliations:** ^1^Department of Ophthalmology & Visual Sciences, University of Texas Medical Branch, Galveston, TX 77555, USA; ^2^Department of Neuroscience, Cell Biology & Anatomy, University of Texas Medical Branch, Galveston, TX 77555, USA; ^3^USANA Health Sciences Inc., Salt Lake City, UT 84120, USA

## Abstract

Signaling pathways mediated by the mechanistic target of rapamycin (mTOR) play key roles in aging and age-related diseases. As a downstream protein of mTOR, transcription factor EB (TFEB) controls lysosome biogenesis and cellular trafficking, processes that are essential for the functions of phagocytic cells like the retinal pigment epithelium (RPE). In the current study, we show that a naturally occurring polyphenolic compound, quercetin, promoted TFEB nuclear translocation and enhanced its transcriptional activity in cultured RPE cells. Activated TFEB facilitated degradation of phagocytosed photoreceptor outer segments. Quercetin is a direct inhibitor of mTOR but did not influence the activity of Akt at the tested concentration range. Our data suggest that the dietary compound quercetin can have beneficial roles in neuronal tissues by improving the functions of the TFEB-lysosome axis and enhancing the capacities of cellular degradation and self-renewal.

## 1. Introduction

Transcription factor EB (TFEB) is a member of the MiTF/TFE protein family that contains a basic helix-loop-helix domain for DNA binding and a leucine-zipper domain for heterodimerization [1]. TFEB controls lysosomal biogenesis and autophagy by positively regulating genes in the Coordinated Lysosomal Expression and Regulation (CLEAR) network [2–5]. Activation of TFEB leads to a coordinated upregulation of CLEAR genes, which collectively improves the efficiency of vesicular trafficking and promotes the eventual substrate degradation at the lysosome. The transcriptional activity and nuclear-cytoplasmic shuttling of TFEB are controlled by mechanistic target of rapamycin (mTOR) complex 1 (mTORC1) [3, 6], which phosphorylates TFEB at its C-terminal serine-rich motif and thereby sequesters TEFB in the cytoplasm [6]. Synthetic chemical inhibitors of mTORC1, such as torin 1 and torin 2, are known activators of TFEB [7–10]. TFEB has been considered a therapeutic target with implications in various human diseases that are associated with defects in autophagy and lysosome-mediated degradation [1, 11]. However, most of the commonly used protein kinase inhibitors such as torins have relatively low substrate specificity and may inhibit other pathways, particularly the Akt-mediated cell survival signaling pathway [12, 13]. Their applications in chronic human degenerative diseases are limited.

Quercetin (3,3′,4′,5,7-pentahydroxyflavone) is a plant-derived polyphenolic compound and is present in a number of dietary components [14]. It is a broad-spectrum protein kinase inhibitor, and a phase I clinical trial of quercetin has demonstrated its tyrosine kinase inhibitory effect [15]. Quercetin has been used as a lead compound for synthesizing derivatives of commonly used kinase inhibitors, such as LY294002 [16]. Whether quercetin influences the activities of mTOR and its downstream proteins like TFEB is not well understood.

The main objective of our current study was to examine the biological effects of quercetin on TFEB in cultured retinal pigment epithelial (RPE) cells. RPE cells provide essential support to the functions of the neurosensory retina [17]. They are phagocytic and have high activity in cellular trafficking and lysosome-mediated degradation processes [17]. Our data show that quercetin dose-dependently activated the transcriptional activity of TFEB and elevated its downstream gene expression. Cells with enhanced TFEB activity had increased autophagy and higher efficiency to degrade phagocytosed photoreceptor outer segments (POS). Quercetin effectively suppressed amino acid-induced mTORC1 activation and likely functioned as a competitive mTOR kinase inhibitor at the ATP-binding motif. These findings provide mechanistic support for the beneficial effects of quercetin as a nutritional supplement to improve the capacity of lysosome-mediated degradation processes in the neuronal tissue.

## 2. Materials and Methods

### 2.1. Materials

Quercetin either was purchased from Sigma-Aldrich (St. Louis, MO, USA) (≥95% HPLC, catalogue number Q4951) or was provided by USANA Health Sciences (Salt Lake City, UT, USA). Cells were treated with 0.5 to 20 *μ*M of quercetin. No difference was observed between the activities of the compounds from the two sources. Torin 1 was purchased from Tocris (Minneapolis, MN). E64d and pepstatin A were purchased from Sigma-Aldrich. A rhodopsin antibody (RET-P1) and TFEB antibody were purchased from Abcam (Cambridge, MA, USA). All other primary antibodies were purchased from Cell Signaling Technology (Danvers, MA, USA). Fluorophore-conjugated secondary antibodies were purchased from Invitrogen (Carlsbad, CA, USA) and LI-COR Biosciences (Lincoln, NE, USA).

### 2.2. Cell Culture

ARPE-19 cells were obtained from the American Type Culture Collection (Manassas, VA, USA) and were cultured in Dulbecco's modified Eagle's medium (DMEM)/Ham's F12 50/50 mix supplemented with 10% fetal bovine serum (Sigma-Aldrich). For quercetin treatment, the compound was dissolved in dimethyl sulfoxide (DMSO) (Sigma-Aldrich) as a stock solution. The final DMSO concentration in the medium of treated cells was less than 0.1%. Cell viability after 20 *μ*M quercetin treatment was assessed by measuring trypan blue exclusion with a Countess automated cell counter (Invitrogen).

### 2.3. Measurement of TFEB Transcriptional Activity by a Reporter Assay

TFEB reporter construct was generated by inserting four tandem copies of the CLEAR motif (5′-GTCACGTGAC-3′) in the pGL3-promoter luciferase reporter vector (Promega, Madison, WI) [18], between KpnI and XhoI sites. The insertion was sequence-verified. For measuring the transcriptional activity of TFEB, ARPE-19 cells in 6-well plates were transiently transfected with 1 *μ*g of the TFEB reporter plasmid per well, using Lipofectamine 2000 Transfection Reagent (Invitrogen). To normalize the transfection efficiency, 30 ng of control reporter construct of Renilla luciferase (pRL-CMV Vector, Promega) was cotransfected [19]. Six hours after transfection, the culture medium was refreshed. One day after transfection, cells were incubated with indicated concentrations (0.5 to 20 *μ*M) of quercetin for additional 16 hr. Afterward, the luciferase activities were measured using a Dual-Luciferase® Reporter Assay System (Promega) following the manufacturer's instructions [19].

### 2.4. Immunofluorescence Staining

For imaging LC3, cultured ARPE-19 cells were seeded on the cover glass and exposed to 10 *μ*M quercetin either in the absence or in the presence of 10 *μ*M chloroquine (CQ). At the end of treatment, cells were rinsed with Tris-buffered saline (TBS) and fixed with 4% paraformaldehyde for 15 min. After permeabilization with methanol for 5 min, the cells were incubated in blocking buffer of 10% FBS and 0.5% Triton X-100 in TBS for 1 hr at room temperature. The cells were then incubated with primary antibodies diluted in the blocking buffer, followed by appropriate fluorophore-conjugated secondary antibodies. After stringent washes, the nuclei were counterstained with 4′,6-diamidino-2-phenylindole (DAPI). The slides were mounted with Fluoro-Gel (Electron Microscopy Service, Hatfield, PA, USA). Fluorescence microscopy was performed using a Zeiss Axio Observer fluorescence microscope equipped with the ApoTome imaging system. The same staining procedures were used for examining TFEB nuclear translocation, in experiment where cells were treated with either 20 or 50 *μ*M quercetin for 2 hr. At least fifty cells were randomly scored per condition per experiment. Image quantification was performed by ImageJ (NIH) [20], by comparing the fluorescence intensity of areas with and without DAPI staining.

### 2.5. RNA Isolation and Quantitative Reserve Transcription PCR (RT-PCR)

Cells were treated with 0.5 to 10 *μ*M quercetin for 16 hr. Total RNA was isolated using TRIzol™ Reagent (Invitrogen) and treated with the DNA-free™ kit (Ambion, Austin, TX, USA) to remove contaminating genomic DNA. The yield and purity of the RNA were determined using a spectrophotometer (NanoDrop ND-1000; Thermo Fisher Scientific, Waltham, MA, USA). Complementary DNA was reversely transcribed from 1 *μ*g of total RNA using oligo(dT)_15_ Primer (Promega). The relative abundance of mRNA expression of CLEAR network genes was determined by quantitative RT-PCR using primers designed with the Universal Probe Library approach (Roche Diagnostics, Indianapolis, IN, USA) [21]. The primers used were as follows: TFEB: 5′-CGG CAG TGC CTG GTA CAT-3′ and 5′-CTG CAT GCG CAA CCC-3′, ATP6V0C: 5′-AGT CCA TCA TCC CAG TGG TC-3′ and 5′-CAG CTG GAG GAA GCT CTT GT-3′, MCOLN1: 5′-AAG GCG ATG GTG TTC TCT TC-3′ and 5′-GCT GCA AGT GGT CAA GAT CC-3′, UVRAG: 5′-CAG AAG GAA TCC CTA AAT GAG C-3′ and 5′-TGC AAC GAA TTG TCA ACT GAG-3′, ATP6V0D2: 5′-ACA AGT CTT ACC TTG AGG CAT TCT-3′ and 5′-TCT GTC GGC CTC AAA CTC A-3′, and PPARGC1a: 5′-TGA GAG GGC CAA GCA AAG-3′ and 5′-ATA AAT CAC ACG GCG CTC TT-3′.

### 2.6. Subcellular Fractionation

Confluent ARPE-19 cells were treated with 20 *μ*M quercetin for 2 hr. At the end of incubation, cells were collected by gentle trypsinization and were washed once with ice-cold TBS. Cytosolic and nuclear extracts were prepared by using the Subcellular Protein Fractionation Kit for Cultured Cells (Thermo Fisher), following the manufacturer's instructions.

### 2.7. Western Blot Analyses

Cells were harvested and lysed in buffer containing a 1 : 1 (*v*/*v*) ratio of CelLytic™ M Cell Lysis Reagent (Sigma-Aldrich) and 2X Laemmli Sample Buffer (Bio-Rad, Hercules, CA, USA), supplemented with 10 mM glycerophosphate, 10 mM pyrophosphate, 1 mM NaF, 1 mM Na_3_VO_4_, and protease inhibitor cocktail [20]. Cell lysates were sonicated, and samples were resolved on SDS-PAGE and transferred to nitrocellulose membranes (Bio-Rad). The membranes were probed with specific primary antibodies followed by appropriate fluorophore-conjugated secondary antibodies. The fluorescent signals were detected by the Odyssey Infrared Imaging System (LI-COR). Detection and quantification of band intensities were performed using Odyssey imaging software version 3.0 (LI-COR) [20].

### 2.8. Measurement of the Turnover Rate of Photoreceptor Outer Segments (POS)

Cultured ARPE-19 cells were incubated with purified porcine POS [22], at a 10 : 1 ratio (POS : cell) for 3 hr. At the end of incubation, unbound POS were removed by washing with phosphate-buffered saline (PBS) containing 1 mM MgCl_2_ and 0.2 mM CaCl_2_ [23]. Cells were kept in the refreshed culture media and chased for up to 4 hr. The amount of rhodopsin in ARPE-19 cells was assessed by Western blot analyses [21]. For experiments on lysosome inhibitors, cells were pretreated for 16 hr with either 10 *μ*M chloroquine or 10 *μ*M E64d and 10 *μ*M pepstatin A, before POS loading.

### 2.9. *In Vitro* Kinase Assay

Inhibition of mTOR kinase activity by quercetin was determined using LanthaScreen™ kinase assay technology from Invitrogen. The LanthaScreen assay is based on time-resolved fluorescence resonance energy transfer (TR-FRET). Kinase reactions were performed in a 10 *μ*L volume in Corning 4513 white 384-well assay plates. The testing compound was diluted into reaction buffer (50 mM HEPES (pH 7.5), 0.01% polysorbate 20, 1 mM EGTA, 2 mM DTT, and 10 mM MnCl2). Kinase (PV4753, mTOR, 114.51 ng/mL final concentration), substrate (GFP-4E-BP1, PV4759, 0.4 *μ*M final concentration), and ATP (PV3227, half Km value was used, 10 *μ*M final concentration) were mixed with inhibitors, and the reaction was allowed to proceed for 1 hr at room temperature. The reaction was stopped by adding antibodies (Tb-anti-p4EBP1, pThr46, PV4757) at a final concentration of 2 mM, diluted in antibody dilution buffer (PV3574) and EDTA (10 mM final concentration). After 30 min incubation with the antibody, the TR-FRET emission ratios were acquired on a Tecan Spark 10M plate reader. A known inhibitor of mTOR, LY294002 (PHZ1144), was used as a positive control and tested using the same conditions.

### 2.10. Statistical Analysis

Data analyses were performed using GraphPad Prism 5. The data given in the text were representatives from at least three independent experiments and were presented as mean ± SEM. *p* < 0.05 (Student's *t*-test or one-way ANOVA) was considered statistically significant.

### 2.11. Computational Modeling

Docking simulation studies of quercetin with mTOR were carried out using the modeling software MOE (Chemical Computing Group Inc., Montreal, Quebec, Canada). Crystal structure of mTOR (PDB ID: 4JT5) was retrieved from the Protein Data Bank (PDB). All molecular visualizations were produced by MOE.

## 3. Results

### 3.1. Induction of TFEB-Mediated Gene Transcription by Quercetin

**Figure 1:**
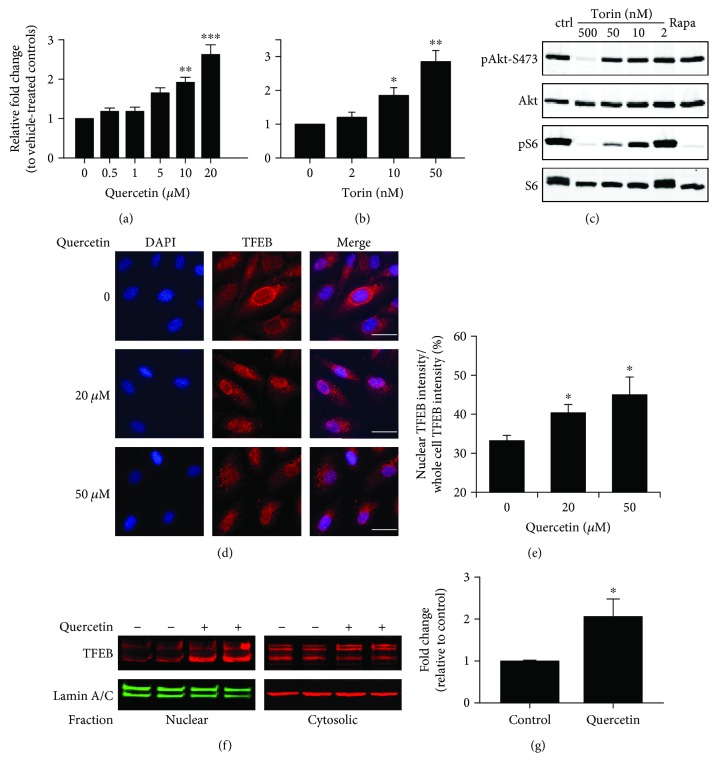
Activation of TFEB by quercetin in cultured ARPE-19 cells. (a, b) Transcriptional activity of TFEB as measured by the luciferase reporter assay. ARPE-19 cells were transfected with the CLEAR-Luc plasmid and measured for dose-dependent responses to quercetin (a) or torin 1 (b) treatment after 16 hr exposure. Data presented are averages from 5 to 6 independent experiments (mean ± SEM). ^∗^*p* < 0.05, ^∗∗^*p* < 0.01, and ^∗∗∗^*p* < 0.001. One-way ANOVA and Dunnett's post hoc test. (c) Western blot showing the dose-dependent effects of torin 1 on Akt and S6 phosphorylation. Cells were treated with the indicated concentrations of torin for 16 hr. The last lane was the sample from cells treated with 20 nM rapamycin (Rapa) for 16 hr. (d) Immunofluorescence staining of TFEB nuclear translocation after 2 h exposure to 20 or 50 *μ*M quercetin. Quantification data are presented in (e). Scale bar: 10 *μ*m. (f) Measurement of TFEB nuclear translocation after subcellular fractionation. RPE cells were treated with 20 *μ*M quercetin for 2 hr, and the amount of TFEB in the nuclear and cytosolic fractions was determined by Western blot analyses. Quantification data are presented in (g). Data presented are averages from 3 independent experiments (mean ± SEM). ^∗^*p* < 0.05. Student's *t*-test.

We used a luciferase reporter assay [24] to screen for nutritional compounds that can potentially activate TFEB. Cultured human ARPE-19 cells were transiently transfected with a reporter plasmid with the CLEAR elements inserted in the enhancer region of the luciferase gene and were treated with various doses of testing compounds for 16 hr. The luciferase activities were measured as an indicator of the transcriptional activity of TFEB. Among the compounds screened, quercetin was identified as a reliable inducer of TFEB. APRE-19 cells exposed to 0.5 to 20 *μ*M quercetin showed a dose-dependent increase in TFEB-driven luciferase activity (Figure 1(a)). At 20 *μ*M, quercetin achieved 2.5-fold induction (1.5–3.3, 95% confidence interval) of the reporter activity. As a validation of the reporter assay, torin 1, which is a well-established mTOR inhibitor [7], was used to treat the ARPE-19 cells. The transcriptional activity of TFEB was elevated by torin 1 (Figure 1(b)), at a concentration range of 2 to 50 nM that suppressed the phosphorylation of S6 but not the phosphorylation of Akt (Figure 1(c)). No sign of cytotoxicity was observed at 20 *μ*M of quercetin. After 48 hr treatment, the viability of vehicle- and quercetin-treated cells was 91.3 ± 2 and 91.3 ± 5.7, respectively (mean ± SD, *N* = 3).

Phosphorylation of TFEB by mTOR leads to its sequestration in the cytoplasm near lysosomes [6]. Upon quercetin treatment, the amount of TFEB protein in the nucleus was increased, as determined by both immunofluorescence staining (Figures 1(d) and 1(e)) and subcellular fractionation (Figures 1(f) and 1(g)). The distribution of TFEB in the cytoplasm, however, was not influenced by quercetin under the experimental conditions.

**Figure 2:**
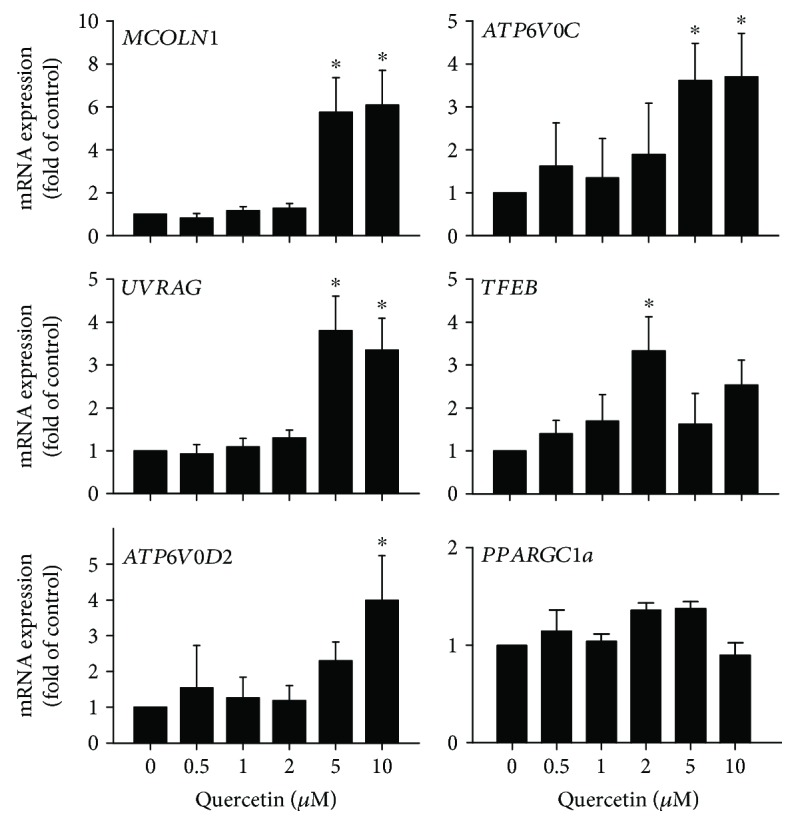
Induction of TFEB downstream genes by quercetin. Dose-dependent upregulation of TFEB in downstream genes in cultured ARPE-19 cells. Cells were treated with the indicated concentration of quercetin for 16 hr and analyzed by quantitative RT-PCR. Data presented are averages from 4 independent experiments (mean ± SEM). ^∗^*p* < 0.05. One-way ANOVA and Dunnett's post hoc test.

The mRNA levels of known TFEB target genes, including the ones involved in cellular vesicular trafficking and lysosome functions [2, 3, 5, 25], were measured in cells treated with 0.5 to 10 *μ*M quercetin (Figure 2). Quercetin treatment at 5 and 10 *μ*M concentrations significantly upregulated *UVRAG*, *MCOLN1*, *ATP6V0C*, and *ATP6V0D2*. The expression of *PPARGC1A* did not respond to quercetin treatment. TFEB itself was upregulated by 2 *μ*M quercetin treatment. Thus, by measuring the transcriptional activity of TFEB, its nuclear translocation, and its downstream gene expression, the data collectively demonstrate that quercetin treatment activates TFEB in the ARPE-19 cells.

### 3.2. Quercetin Enhanced TFEB-Mediated Cellular Degradation Capacity in the RPE

**Figure 3:**
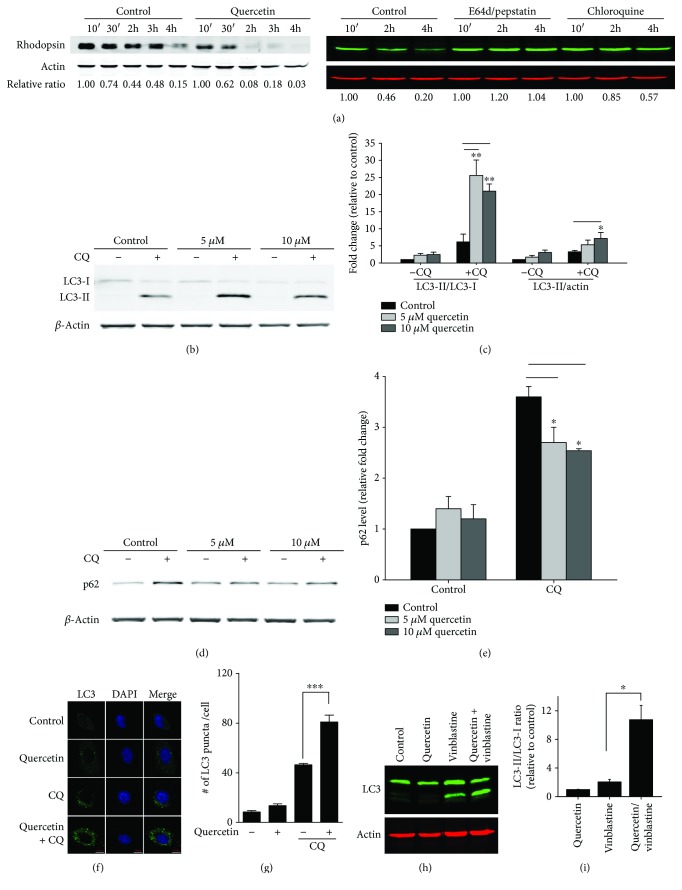
Enhancing RPE cell degradation capacity by quercetin. (a) Measurement of the POS turnover rate. ARPE-19 cells were treated with 10 *μ*M quercetin, or vehicle control, for 16 hr and subsequently were loaded with purified POS (5 : 1 ratio, POS : RPE) for 3 hr. After stringent washes, the rates of degradation of engulfed POS were measured by Western blot analyses of rhodopsin. To study the effects of lysosome inhibitors, cells were treated with 10 *μ*M of E64d and pepstatin A or 10 *μ*M CQ for 16 hr and then loaded with POS. (b) Effects of quercetin on LC3 lipidation. Cells were treated with the indicated concentration of quercetin, with or without 10 *μ*M CQ, for 16 hr. Quantification data are presented in (c). (d, e) Western blot analyses of p62 protein in ARPE-19 cells treated with quercetin alone or with CQ. (f) Immunostaining of LC3 punctum formation after quercetin and CQ treatment; quantification data are presented in (g). (h, i) Effects of quercetin and vinblastine treatments on LC3 lipidation. Data presented are averages from 3–5 independent experiments (mean ± SEM). ^∗^*p* < 0.05, ^∗∗^*p* < 0.01, and ^∗∗∗^*p* < 0.001. One-way ANOVA and Dunnett's post hoc test.

A specialized function of the RPE is phagocytosis of shed POS from photoreceptor neurons [17]. The turnover and recycling processes of POS are part of the visual cycle and are critical for retinal health and function [26]. Protein components of the internalized POS, such as rhodopsin, are eventually degraded in lysosomes. Inhibiting lysosome proteolysis with E64d and pepstatin effectively protects rhodopsin from degradation [27]. Because TFEB controls cellular trafficking and lysosome function, we examined whether ARPE-19 cells treated with quercetin had increased degradation capacity for POS. Cells were first loaded with purified porcine POS for 3 hours. Afterward, the unbound POS were removed and the remaining POS in cells were monitored by measuring the level of rhodopsin, the major protein component that is unique to POS. As shown in Figure 3(a), cells treated with 10 *μ*M quercetin had accelerated POS degradation. Two hours after chasing, most of the rhodopsin associated with ingested POS were degraded in quercetin-treated RPE cells, while control cells still had a notable amount of rhodopsin between 2 and 3 hr. POS degradation in the ARPE cells is dependent on lysosome functions. Inhibitors of lysosome acid proteases, chloroquine (CQ), E64d, and pepstatin A, effectively suppressed the turnover of ingested POS (Figure 3(a)).

Next, we examined the effects of quercetin on markers of autophagy. RPE cells are highly efficient in autophagy, and CQ treatment is required to visualize autophagosome and LC3-II under the experimental conditions (Figure 3(b)). We found that in the presence of CQ, 5 and 10 *μ*M quercetin treatment increased the level of LC3-II, the lipidated form of LC3 and a marker protein of autophagosomes [28, 29], as compared to CQ treatment alone (Figures 3(b) and 3(c)). The ratio of LC3-II/LC3-I was also increased (Figure 3(c)), while the level of autophagy substrate protein p62 was decreased (Figures 3(d) and 3(e)). Both changes after exposure to quercetin indicated the enhanced autophagy. When examined by immunofluorescent staining, the number of LC3-positive puncta was markedly increased by quercetin in the presence of CQ (Figures 3(f) and 3(g)). Similar results were obtained when cells were treated with vinblastine, a compound that disrupts autophagic trafficking on microtubules (Figures 3(h) and 3(i)) [30]. Thus, with multiple independent measurements, we showed that quercetin treatment can elevate the cellular trafficking and degradation capacity in the RPE.

### 3.3. Inhibition of mTOR Kinase Activity by Quercetin

**Figure 4:**
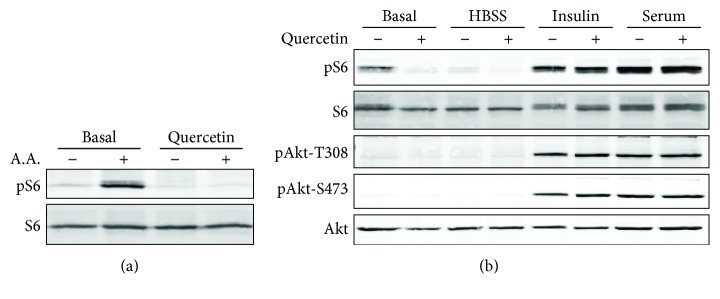
Effects of quercetin on mTOR activity in RPE cells. Cells were pretreated with 10 *μ*M quercetin for 16 hr and stimulated with either amino acids (a), insulin, or serum (b) for 30 min. Phosphorylation of S6 and Akt was measured by Western blot analyses as indicators of mTOR kinase activity. Amino acids (almost all) used were a mixture of essential amino acids used in Minimum Essential Medium (MEM), including glutamine. Basal: cells incubated in serum-free medium.

A key upstream regulator of TFEB is mTORC1 [6, 9]. In RPE cells, mTORC1 can be activated by various stimuli including nutrient and growth factors [21]. In ARPE-19 cells treated with 10 *μ*M quercetin, there was a selective suppression of amino acid-induced mTORC1 activation, as measured by the phosphorylation status of its downstream ribosome protein S6 (Figure 4(a)). Insulin- or serum-induced mTOR activation was not influenced by quercetin at this dosage (Figure 4(b)). Furthermore, quercetin did not alter the phosphorylation of Akt (Figure 4(b)), suggesting that it did not inhibit mTOR complex 2 that phosphorylates Akt at serine 473 [31]. Phosphorylation of Thr308, an indicator of Akt activity [32, 33], was not influenced either.

**Figure 5:**
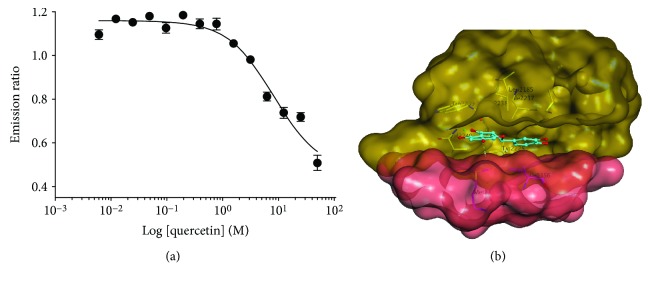
Quercetin as a direct inhibitor of mTOR kinase. (a) *In vitro* kinase assay showing a dose-dependent inhibition of mTOR activity by quercetin. (b) Structural modeling of interactions of quercetin with the ATP-binding pocket of mTOR.

To assess whether quercetin is a direct inhibitor of mTOR, we performed an *in vitro* kinase assay with purified mTOR protein. As shown in Figure 5(a), quercetin suppressed the kinase activity of mTOR in a dose-dependent way, and the IC50 was 7.8 *μ*M, which is consistent with the concentration range for the cell-based assays (Figure 1).

To further understand the mechanism of inhibition at the structural level, computer-based molecular docking modeling was utilized to explore the interaction of quercetin with mTOR at the ATP-binding site. The modeling revealed that the most apparent interactions were the two hydrogen bonds between the benzene rings of quercetin and Tyr225 and Met2345 of mTOR. The carbonyl group of quercetin serves as a potential backbone acceptor for the interaction with Val2240, and the methoxyl group as a backbone donor for Gly2238 (Figure 5(b)).

## 4. Discussion

In the current study, we investigated the effects of a naturally occurring polyphenolic compound, quercetin, on the functions of the transcription factor TFEB. Using a combination of immunostaining, subcellular fractionation, luciferase reporter assay, and quantitative RT-PCR approaches, we demonstrate that quercetin exposure elevated the transcriptional activity of TFEB (Figure 1(a)), promoted its nuclear translocation (Figures 1(d) and 1(f)), and upregulated the downstream gene expression (Figure 2). Consistent with our findings, a structurally related polyphenolic compound, fisetin, was recently reported to activate TFEB and induce autophagy [34, 35].

Nutritional supplementation has been viewed as a promising interventional approach for delaying the progression of age-related neurodegenerative disorders [36, 37]. Quercetin was previously reported as an effective treatment against tauopathy and *β*-amyloidosis in a mouse model of Alzheimer's disease and preserved cognitive functions [38]. A number of protective mechanisms have been proposed, and enhancing TFEB-mediated autophagy and lysosome functions likely contributes to the beneficial effects of quercetin in the central nervous system. The retina and RPE are highly active in metabolism and are susceptible to proteolytic and ER stress [39]. Similar to the effects in the brain, enhancing the degradation capacity of RPE can be beneficial for the improvement of the health of the retina under chronic disease conditions. Whether quercetin can exert long-term protective effects can be explored by future *in vivo* studies.

Quercetin was used as a model compound to develop kinase inhibitors [16]. Our data from cell-based assays (Figure 4) and *in vitro* kinase assay (Figure 5(a)) demonstrated that quercetin is a direct inhibitor of mTOR. Recently, quercetin has been reported as being involved in Akt-mTOR and HIF-1*α* signaling [40]. Noticeably, quercetin did not inhibit Akt phosphorylation in RPE cells at the dose range that effectively suppressed mTORC1 (Figure 4(b)). The relative substrate selectivity is a unique advantage of quercetin. Rapamycin or its analogs are prototypical inhibitors of mTORC1, but whether they can effectively activate TFEB remains unclear [8]. The second-generation mTOR inhibitors, torin 1 and torin 2, are highly potent and have well-established roles in TFEB activation [8] (Figure 1(b)). However, they can also have potent inhibitory effects on the mTORC2 pathway, and the suppression on the Akt activity leads to severe cytotoxicity [12, 13].

The limitation of our study is that the concentration of quercetin that effectively inhibits mTORC1 is relatively higher than the reported blood concentration of quercetin from previous human clinical trials. For example, one study showed that healthy volunteers who had taken 1 gram of quercetin per day for 28 days reached about 1.5 *μ*M quercetin in the plasma [41]. Future studies on the pharmacokinetic and pharmacodynamic properties can be performed to define whether quercetin can be pursued as a nutritional supplement for intervening human diseases. The tissue concentration can be different from the blood quercetin concentration, and the dose and safety ranges will have to be further examined.

In summary, our work showed that quercetin is a naturally occurring compound that exerts beneficial effects on the central nervous system via TFEB-dependent mechanisms. Genes in the CLEAR network encode proteins that are involved in trafficking, autophagy, and lysosome degradation. Other than the translational implications, further exploring the genes that function downstream of TFEB and control RPE trafficking and lysosomal degradation can reveal novel mechanistic information of RPE cell biology.

## Data Availability

The data used to support the findings of this study are included within the article.
